# Interferon-γ/Interleukin-27 Axis Induces Programmed Death Ligand 1 Expression in Monocyte-Derived Dendritic Cells and Restores Immune Tolerance in Central Nervous System Autoimmunity

**DOI:** 10.3389/fimmu.2020.576752

**Published:** 2020-10-26

**Authors:** Giacomo Casella, Javad Rasouli, Rodolfo Thome, Hélène C. Descamps, Asrita Vattikonda, Larissa Ishikawa, Alexandra Boehm, Daniel Hwang, Weifeng Zhang, Dan Xiao, Jeongho Park, Guang-Xian Zhang, Jorge I. Alvarez, Abdolmohamad Rostami, Bogoljub Ciric

**Affiliations:** ^1^Department of Neurology, Thomas Jefferson University, Philadelphia, PA, United States; ^2^Department of Microbiology, Perelman School of Medicine, University of Pennsylvania, Philadelphia, PA, United States; ^3^Department of Pathobiology, School of Veterinary Medicine, University of Pennsylvania, Philadelphia, PA, United States; ^4^College of Veterinary Medicine & Institute of Veterinary Science, Kangwon National University, Chuncheon, South Korea

**Keywords:** peripheral tolerance, monocytes, experimental autoimmune encephalitis, PD-L1, cytokines

## Abstract

Antigen (Ag)-specific tolerance induction by intravenous (i. v.) injection of high-dose auto-Ags has been explored for therapy of autoimmune diseases, including multiple sclerosis (MS). It is thought that the advantage of such Ag-specific therapy over non-specific immunomodulatory treatments would be selective suppression of a pathogenic immune response without impairing systemic immunity, thus avoiding adverse effects of immunosuppression. Auto-Ag i.v. tolerance induction has been extensively studied in experimental autoimmune encephalomyelitis (EAE), an animal model of MS, and limited clinical trials demonstrated that it is safe and beneficial to a subset of MS patients. Nonetheless, the mechanisms of i.v. tolerance induction are incompletely understood, hampering the development of better approaches and their clinical application. Here, we describe a pathway whereby auto-Ag i.v. injected into mice with ongoing clinical EAE induces interferon-gamma (IFN-γ) secretion by auto-Ag-specific CD4^+^ T cells, triggering interleukin (IL)-27 production by conventional dendritic cells type 1 (cDC1). IL-27 then, *via* signal transducer and activator of transcription 3 activation, induces programmed death ligand 1 (PD-L1) expression by monocyte-derived dendritic cells (moDCs) in the central nervous system of mice with EAE. PD-L1 interaction with programmed cell death protein 1 on pathogenic CD4^+^ T cells leads to their apoptosis/anergy, resulting in disease amelioration. These findings identify a key role of the IFN-γ/IL-27/PD-L1 axis, involving T cells/cDC1/moDCs in the induction of i.v. tolerance.

## Introduction

Autoimmune diseases develop due to a break in immune tolerance toward certain auto-antigens (auto-Ags). It follows that the healthy state could be achieved by restoring peripheral immune tolerance toward those auto-Ags. Devising therapies based on the restoration of Ag-specific immune tolerance induction has been a long-standing goal for treatment of autoimmune diseases, including multiple sclerosis (MS) (1). One such approach relies on intravenous (i.v.) injection of free myelin-derived auto-Ags that are targets of autoimmune response. This approach, and variants thereof, have been proven beneficial in experimental autoimmune encephalomyelitis (EAE), an animal model of MS (1). Some clinical trials have confirmed that repeatedly i.v. injecting large doses of myelin auto-Ag can be safe and beneficial to a subset of MS patients (1). In comparison with non-specific immunomodulatory therapies currently in use, the principal advantage of Ag-specific therapy would be that it suppresses harmful autoimmune response while sparing the rest of the immune system. This would eliminate side effects and adverse events due to systemic immunosuppression caused by non-specific immunomodulation.

Even though certain key players in i.v. tolerance induction, such as interleukin (IL)-10, IL-27, and programmed death ligand 1 (PD-L1), have been identified (2, 3), its complete mechanisms have not been elucidated. This lack of specific knowledge also includes the cell types involved, sequence of their interactions, and relative relevance of the periphery vs. the central nervous system (CNS) in tolerance induction and maintenance over time. A more thorough understanding of these mechanisms will be helpful in developing better Ag-specific therapies for MS and possibly other autoimmune diseases.

Consistent with the suppressive role of IL-10 in EAE development (4), i.v. tolerance induction in EAE requires IL-10. Tolerization by i.v. injection of an auto-Ag elicits IL-10 production, and blockade of IL-10 signaling precludes tolerance induction (2, 5, 6).

The lack of IL-27 signaling leads to the development of more severe EAE (7), and treatment with recombinant IL-27 suppresses EAE (8–10), demonstrating its anti-inflammatory role in EAE. The anti-inflammatory effects of IL-27 encompass inhibition of Th17 cell development; suppression of granulocyte-macrophage colony-stimulating factor (GM-CSF) expression; induction of PD-L1, CD39, and IL-10 expression; and enhancement of Treg development and function (8–10). We have shown that IL-27 is necessary for induction of i.v. tolerance in EAE (3); in particular, IL-27 signaling in DCs was required for tolerance induction, whereas its signaling in T cells was not. IL-27-dependent tolerance induction relied on cooperation of distinct subsets of spleen DCs with the ability to induce T cell-derived IL-10 and interferon-gamma (IFN-γ) (3).

Programmed cell death protein 1 (PD-1) and its ligands, PD-L1 and PD-L2, regulate the balance between T cell activation and immune tolerance (11, 12). The majority of CD4^+^ T cells in the CNS of mice with EAE express PD-1, while PD-L1 and PD-L2 are differentially expressed by populations of Ag-presenting cells (APC) (13). PD-1^−/−^ and PD-L1^−/−^ mice develop atypically severe EAE, with enhanced T cell proliferation and increased production of inflammatory cytokines (14, 15). Genetic deficiency in PD-L2 did not lead to more severe EAE (14), suggesting that PD-L1 is a dominant inhibitory PD-1 ligand in EAE development. In contrast, blockade of PD-L2, but not PD-L1, in advanced EAE in C57BL/6 mice led to worsening of disease, indicating that these two PD-1 ligands, or possibly cell types that express them, have distinct roles in regulating different stages of EAE (2, 16, 17). In regard to i.v. tolerance induction in EAE, it has been shown that tolerization induces PD-L1 expression by APCs and that PD-1 blockade abrogates tolerance induction (2, 18).

IFN-γ is a cytokine released by almost all activated immune cells, with natural killer (NK) and T cells being its major sources (19). Although IFN-γ has been traditionally considered a pro-inflammatory cytokine, it is now clear that IFN-γ also has prominent anti-inflammatory roles that balance its possibly damaging inflammatory effects (20). Numerous studies have firmly established that IFN-γ suppresses EAE; mice lacking IFN-γ signaling develop severe EAE, and mouse strains resistant to EAE become susceptible (21–23). Consistent with this, IFN-γ production by myelin-specific CD4^+^ T cells is not required for their encephalitogenicity (24), IFN-γ-deficient CD4^+^ T cells could be notably more pathogenic than their IFN-γ-sufficient counterparts (25), and IFN-γ production by encephalitogenic T cells in the CNS is required for recovery from EAE (26). Further, an increase in IFN-γ levels in the CNS of mice with EAE leads to disease suppression (27, 28). Taken together, these findings indicate that IFN-γ could be important in EAE suppression by i.v. tolerance induction as well, a possibility that has not been explored.

Here we show that i.v. administration of auto-Ag (free encephalitogenic peptide) halts EAE progression by inducing PD-L1 expression in CNS monocyte-derived dendritic cells (moDCs) *via* an IFN-γ/IL-27-dependent mechanism. Blockade of PD-L1, but not PD-L2, or the lack of PD-1 in CD4^+^ T cells precluded i.v. tolerance induction. The lack of IFN-γ in CD4^+^ T cells, or IFN-γR in conventional DCs type 1 (cDC1), abrogated IL-27 production and PD-L1 expression by moDCs. Collectively, our data reveal a mechanism of Ag-dependent induction of PD-L1 expression in moDCs that in turn suppresses Ag-specific Th cell responses and ameliorates CNS autoimmunity.

## Materials and Methods

### Mice

C57BL/6, B6.Ly5.1 (CD45.1^+^), RAG1^−/−^, PD-1^−/−^, 2D2, Zbtb46-iDTR, IFN-γ^−/−^, IFN-γRα^−/−^, GREAT (IFN-γ reporter), *Ccr2*^−/−^, *Wsx*^−/−^, and *Stat3*^mut^ mice were purchased from The Jackson Laboratory (Bar Harbor, ME, USA). IL-27p28 reporter mice were a gift of Dr. Ross M. Kedl (University of Colorado). Mice were kept in specific pathogen-free conditions with a maximum of 5 mice per cage, in 12/12 h of light/dark cycles and food *ad libitum* throughout the experimental procedures. Every effort was made to minimize suffering of mice. Experimental protocols using mice were approved by the Institutional Animal Care and Use Committee of Thomas Jefferson University.

### Generation of BMDCs

STAT1^−/−^, STAT3^−/−^, and WT BMDCs were generated according to a previously described protocol (3). Briefly, BM cells were seeded at 2 × 10^6^ cells/mL in Petri dish in complete IMDM supplemented with 100 ng/mL of recombinant mouse Flt-3 (R&D Systems, Minneapolis, MN, USA). Culture medium was changed every 3 days. Maturation of the DCs was induced with LPS (100 ng/mL) for 16 h. At day 9 after starting the culture, DCs were enriched by anti-Flt-3-biotin Ab and anti-biotin microbeads (Miltenyi Biotec, CA, USA), and CD11c^+^MHCII^+^ cells were then FACS-sorted.

IFN-γRα^−/−^ and WT cDCs were generated from BM cells following a protocol described in (29) with slight modifications.

### Ag-presentation Assays

Naive CD4^+^ T cells from spleens of 2D2 mice were isolated using magnetic beads (Naive CD4^+^ T cell isolation kit, Miltenyi Biotec, CA, USA). 2 × 10^5^ naive CD4^+^ T cells were added to each well of the cell culture plate containing moDCs, cDC1, or BMDCs (ratio of 1 DC: 10 T cells) and plates were incubated at 37°C in the presence of MOG_35−55_ peptide (20 μg/mL) and anti-PD-L1 MAb (1 μg/mL; clone 10F.9G2, BioXCell). Cells were collected after 72 h and analyzed by flow cytometry, while cytokine concentrations in culture supernatants were measured by ELISA.

### EAE and i.v. Tolerance Induction

Anesthetized mice were subcutaneously injected with 200 μL of an emulsion containing 200 μg of MOG_35−55_ peptide (MEVGWYRSPFSRVVHLYRNGK, Genscript, NJ, USA) in PBS and equal volume of Complete Freund's adjuvant supplemented with 10 mg/mL of heat-killed *Mycobacterium tuberculosis* H37Ra. Additionally, mice were intraperitoneal (i.p.) injected with 200 ng of pertussis toxin at immunization time and 48 h later. Mice were weighed and scored for clinical signs daily. Clinical assessment of EAE was performed according to the following scoring criteria: 0, healthy; 1, limp tail; 2, ataxia and/or paresis of hindlimbs; 3, paralysis of hindlimbs and/or paresis of forelimbs; 4, tetraparalysis; and 5, moribund or death (30).

i.v. tolerance was induced in mice after onset of clinical disease by injections of 200 μg MOG_35−55_ in PBS every third day, 3 times in total. Control mice received PBS only (3).

### Bone Marrow Chimeras

B6.Ly5.1 (CD45.1^+^) congenic mice were lethally irradiated with 2 × 2.5 Gy with an 8 h interval between irradiation and were then i.v. injected with 5 × 10^6^ CD45.2^+^ BM cells from WT, or Zbtb46-DTR donors. Recipient mice were in other experiments reconstituted with 1:1 mixture (total 1 × 10^7^ cells) of BM cells from *Wsx*^−/−^ and *Ccr2*^−/−^ mice, or with mixture of BM cells from *Stat3*^mut^ and *Ccr2*^−/−^ mice. Mice were allowed to reconstitute for 6–8 weeks prior to use.

### DT Ablation

Diphtheria toxin (DTX; Sigma-Aldrich) was administered i.p. at 1 μg/20 g of mouse weight in 200 μl of PBS, 1 day before i.v. injection of MOG_35−55_. Mice received three injections of DTX once every 3 days.

### PD-L1, PD-L2, and IFN-γ Blockade

Mice with EAE were i.p. injected with 200 μg/mouse of αPD-L1 MAb (clone 10F.9G2, BioXCell), or with 200 μg/mouse of αPD-L2 MAb (clone TY25, BioXCell), or with 150 μg/mouse of αIFN-γ MAb (clone R4-6A2, BioXCell) 1 day before i.v. injection of MOG_35−55_. Mice received two MAb injections, 3 days apart in each treatment.

### Ag-Specific Recall Response

Spleens of mice with EAE were dissociated through a 70 μm strainer to prepare single-cell suspensions in complete IMDM, containing 10% heat-inactivated fetal bovine serum, penicillin (100 U), streptomycin (10 μg/mL), L-glutamine (0.3 mg/mL), and 2-mercaptoethanol (55 μM). After treatment with RBC lysis buffer (Biolegend, CA, USA), cells were extensively washed with complete IMDM by centrifugation at 1,300 rpm for 5 min at 4°C and the cell density was adjusted to 2 × 10^6^ /mL. One hundred microliter of adjusted cell suspension was added to each well of a 96-well plate. MOG_35−55_ was added to a final concentration of 20 μg/mL. Cells were incubated at 37°C for 3 days. As negative control, cells were cultured without MOG_35−55_. Cell culture supernatants were collected and stored at −20°C until use, and cells were analyzed for proliferation and cytokine production by flow cytometry.

### Reconstitution of WT and RAG1^–/–^ Mice

WT mice with EAE received i.v. 2 × 10^6^ FACS-sorted CD11b^+^ CD11c^+^ Ly6c^high^ MHCII^+^ cells from the CNS of WT mice with EAE previously i.v. injected with MOG_35−55_ or PBS. CD45.1^+^ mice reconstituted with CD45.2^+^ BM cells from WT, or Zbtb46-iDTR donors, received i.v. 2 × 10^6^ of *in vitro* Flt-3-differentiated cDCs. RAG1^−/−^ mice were i.v. reconstituted with 5 × 10^6^ magnetic bead-isolated total CD4^+^ T cells from spleens of WT, PD1^−/−^, or IFN-γ^−/−^ mice. After 72 h of adoptive transfer, mice were immunized for EAE induction.

### Isolation of CNS Infiltrating Leukocytes

Brain and spinal cord tissues were incubated for 30 min at 37°C with 0.4 mg/mL type IV collagenase (Sigma-Aldrich) and dissociated by passing through a 19-gouge needle. Cells were enriched by centrifugation on a Percoll gradient as previously described (31).

### Flow Cytometry and Cell Sorting

Flow cytometry was performed using a FACSaria II (Becton Dickinson) and analyzed with FlowJo software (Tree Star). Fluorochrome-conjugated MAbs specific for: CD45 (clone 30-F11), CD45.1 (A20), CD11b (M1/70), CD3 (17A2), CD8α (53-6.7) CD4 (RM4-5), CD19 (1D3/CD19), CD11c (N418), CD26 (H194-112), CD88 (20/70), CD172α (P84), PDCA1 (927), Ly6c (AL-21), F4/80 (MB8), Ly6g (1A8), MHC-II (M5/114.15.2), PD-1 (29F.1A12), PD-L1 (10F.9G2), PD-L2 (TY25), Caspase 3, and Annexin V were purchased either from BD Biosciences, R&D, Biolegend, Santa Cruz, or Abcam.

For intracellular staining, cells were stimulated for 4 h with phorbol 12-myristate 13-acetate (PMA; 50 ng/ml, Sigma-Aldrich) and ionomycin (500 ng/ml, Sigma-Aldrich) in the presence of GolgiPlug (1:1,000, BD Pharmigen), permeabilized using a Cytofix/Cytoperm Plus kit (BD Bioscience) and stained with the following fluochrome-conjugated MAbs: GM-CSF (MP1-22E9), IL-17A (TC11-18H10.1), and IFN-γ (XMG1.2) from Biolegend and BD Pharmingen. Dead cells were excluded using L/D stain (BD Pharmingen). Data were acquired on a FACSAria Fusion (BD Biosciences) and analyzed using FlowJo software (TreeStar).

### ELISA

Supernatants from cell cultures were kept at −20°C until use. Cytokine concentrations in culture supernatants were measured with sandwich enzyme-linked immunosorbent assay (ELISA) using commercial kits, following the manufacturer's recommendation (R&D Systems, Minneapolis, MN, USA).

### qPCR

Total RNA was extracted from moDCs, CD4^+^ T cells, and cDC1 with RNeasy Mini Kit (Qiagen), whereas from total CNS and spleen with Trizol (Invitrogen). Genomic DNA was removed by treatment with DNAse I type (Qiagen). cDNA synthesis was performed using ThermoscriptTM RT-PCR system (Invitrogen). Apoptosis (cat# 4413255), Jak/Stat signaling (cat# 4391524) arrays, *Il27ra*, (Mm00497259_m1), and *Gapdh* (4352339E). mRNA levels were measured by real-time RT-PCR (Applied Biosystems, Invitrogen). The 2–ΔΔCT method was used to calculate relative changes in gene expression (32).

### Statistical Analysis

Statistical analysis was performed by GraphPad Prism 8 software. Statistical evaluations are expressed as mean ± s.d. or mean ± s.e.m., as appropriate. Results were analyzed using Two- or One-way ANOVA and posttested with Bonferroni, and with unpaired, two-tailed Student's *t*-test. Statistical significance was ranked ^*^p < 0.05; ^**^p < 0.001; ^***^p < 0.0001; ^****^p < 0.00001.

## Results

### Intravenous Tolerance Induction in Experimental Autoimmune Encephalomyelitis Is Dependent on Programmed Death Ligand 1 and Programmed Cell Death Protein 1, but Not on Programmed Death Ligand 2

**Figure 1:**
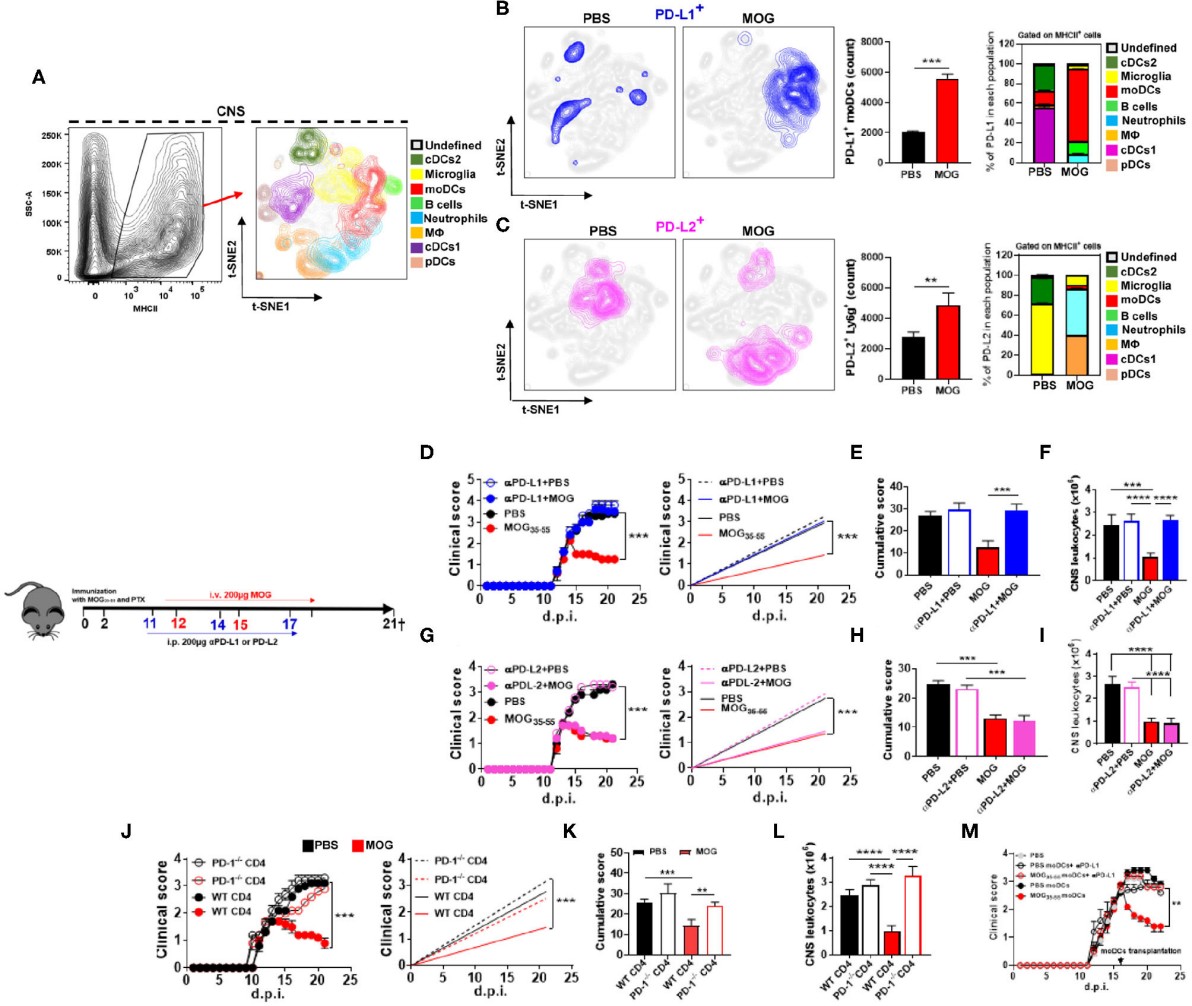
Intravenous administration of myelin oligodendrocyte glycoprotein (MOG)_35−55_ induces programmed death ligand 1 (PD-L1) expression in central nervous system (CNS) monocyte-derived dendritic cells (moDCs) and suppresses experimental autoimmune encephalomyelitis (EAE) in a programmed cell death protein 1 (PD-1)/PD-L1 manner. C57BL/6 wild-type (WT) mice (*n* = 10/group each experiment) were immunized with MOG_35−55_ for EAE induction and starting from disease onset i.v. injected with 200 μg of MOG_35−55_ in PBS every 3 days. Mice were sacrificed 21 days post immunization (d.p.i.) and CD45^+^ MHCII^+^ cells from the CNS were analyzed by flow cytometry. **(A)** Eight populations were identified: microglia (CD11b^+^ CD45^low^), monocytes (CD11b^+^ CD11c^+^ Ly6c^high^ Ly6g^−^), macrophages (CD11b^+^ Ly6c^low^ Ly6g^−^ F4/80^high^), cDC1 (CD11b^+^ CD11c^+^ Ly6c^med^ CD26^+^), cDC2 (CD11b^+^ CD11c^+^ Ly6c^med^ CD172α^+^), plasmacytoid DCs (pDCs; Lin^−^ PDCA1^high^), and B cells (CD19^+^). **(B,C)** t-SNE graphs showing the expression and percentage of PD-L1^+^ **(B)** and PD-L2 **(C)** cells from the CNS of PBS- or MOG_35−55_-treated EAE mice. **(D)** Mice with EAE (*n* = 5–7/group in each experiment) were i.p. injected with blocking anti-PD-L1 MAb (200 μg/mouse/injection; clone 10F.9G2), or isotype control MAb, on 11, 14, and 17 d.p.i. MOG_35−55_ or PBS was i.v. injected on 12, 15, and 18 d.p.i. **(E)** Cumulative disease score for mice described in **(D)**. **(F)** Mice described in **(D)** were sacrificed at day 21 p.i. and the numbers of CD45^+^ leukocytes obtained from their CNS were determined by flow cytometry. **(G)** Mice with EAE (*n* = 5–7/group in each experiment) were i.p. injected with blocking anti-PD-L2 MAb (200 μg/mouse/injection; clone TY25), or isotype control MAb, as described in **(D)**. **(H)** Cumulative disease score for mice described in **(G)**. **(I)** Mice described in **(G)** were sacrificed at day 21 p.i. and the numbers of CD45^+^ leukocytes obtained from their CNS were determined by flow cytometry. **(J)** RAG1^−/−^ mice were reconstituted with 5 × 10^6^ total CD4^+^ T cells from WT or PD-1^−/−^ mice (*n* = 10/group in each experiment); 72 h post reconstitution, recipient mice were immunized for EAE induction and MOG_35−55_ or PBS was injected i.v. three times, starting from EAE onset. **(K)** Cumulative disease score for mice described in **(J)**. **(L)** Mice described in **(J)** were sacrificed at day 21 p.i. and the numbers of CD45^+^ leukocytes obtained from their CNS were determined by flow cytometry. **(M)** Recipient WT mice with EAE were transplanted at the peak of disease with 2 × 10^6^ moDCs from the CNS of donor mice with EAE that were previously i.v. tolerized with MOG_35−55_ or PBS (*n* = 5/group in each experiment). Groups of recipient mice with EAE received moDCs that were pretreated for 1 h with anti-PD-L1 MAb (1 μg/ml). All data are representative of at least two experiments and symbols depict mean ± SEM. Analyses between two groups were carried out by Student's *t-*test and between four groups by one-way ANOVA with Bonferroni post-test **(E,F,H,I,K,L)**. EAE experiments were analyzed by two-way ANOVA with Bonferroni's multiple comparison. Values of ^**^P < 0.001, ***P < 0.0001, and ****P < 0.00001 were considered significant.

We and others have reported that i.v. delivery of auto-Ag in mice with EAE induces expression of PD-L1 by APCs (2, 3). Given the importance of PD-1 and its ligands in immune tolerance, we investigated their role in i.v. tolerance induction in EAE. Mice were immunized with myelin oligodendrocyte glycoprotein (MOG)_35−55_ for EAE induction and i.v. injected with 200 μg of MOG_35−55_ in phosphate-buffered saline (PBS), or with PBS, at the onset of clinical disease; two more doses of MOG_35−55_ and PBS were injected 3 days apart (Supplementary Figures 1A,B). Mice were sacrificed at 21 days post immunization (d.p.i.), and cells isolated from their CNS were analyzed by flow cytometry. t-distributed stochastic neighbor embedding (t-SNE) analysis (33) identified eight populations of CD45^+^ MHCII^+^ cells: cDC1, cDC2, microglia, plasmacytoid DCs (pDCs), moDCs, macrophages (MΦ), neutrophils, and B cells (Figure 1A). MOG_35−55_-treated mice had increased numbers of PD-L1^+^ and PD-L2^+^ cells compared with PBS-treated mice. Next, we investigated which of these cells expressed PD-L1 and PD-L2. moDCs (75%) were the bulk of PD-L1^+^ cells, whereas in control mice, cDC1 (58%) and cDC2 (33%) were the most abundant PD-L1^+^ cells (Figure 1B). In mice that received MOG_35−55_, PD-L2 was mostly expressed by neutrophils (50%) and MΦ (35%), whereas in control mice, PD-L2 was expressed by microglia (64%) and cDC2 (34%) (Figure 1C). These data show that i.v. delivery of auto-Ag induces a robust PD-L1 expression by moDCs and PD-L2 expression by neutrophils and MΦ. We also found increased numbers of PD-L1^+^ and PD-L2^+^ cells among lymph node and splenic cells of MOG_35−55_-tolerized mice (data not shown).

We then tested the role of PD-L1 and PD-L2 in tolerance induction by i.p. injecting anti-PD-L1 or anti-PD-L2 MAbs 24 h before i.v. injecting MOG_35−55_. Treatment with anti-PD-L1 MAb precluded tolerance induction (Figures 1D,E) and led to an increase in the number of leukocytes in the CNS (Figure 1F), whereas treatment with anti-PD-L2 MAb did not have an effect (Figures 1G–I). Further, we transplanted PD-1^−/−^ or wild-type (WT) CD4^+^ T cells into RAG1^−/−^ mice and induced EAE in them. MOG_35−55_/i.v. treatment suppressed EAE in mice with WT CD4^+^ T cells, but not in mice with PD-1^−/−^ CD4^+^ T cells (Figures 1J–L). These data demonstrate that PD-L1 and PD-1 are critical for i.v. tolerance induction in ongoing EAE, whereas PD-L2 does not play a role.

### Monocyte-Derived Dendritic Cells From the Central Nervous System of Tolerized Mice With Experimental Autoimmune Encephalomyelitis Are Suppressive via Programmed Death Ligand 1

Given that tolerized mice had increased numbers of apoptotic annexin V^+^ CD4^+^ T cells (Supplementary Figures 1C,D) and PD-L1^+^ moDCs in the CNS (Figure 1B), we investigated a correlation between their numbers. There was a robust positive correlation of apoptotic CD4^+^ T cells with PD-L1^+^ moDCs (Supplementary Figure 1E), but not with PD-L2^+^ moDCs (Supplementary Figure 1F) or PD-L2^+^ neutrophils (data not shown). This suggests that PD-L1^+^ moDCs mediate apoptosis of T cells.

We next evaluated the effect of moDCs on myelin-specific CD4^+^ T cells *ex vivo*. MHCII^+^ Ly6c^high^ moDCs were sorted from the CNS of mice with EAE treated with MOG_35−55_/i.v. and co-cultured with naive CD4^+^ T cells expressing a transgenic T cell receptor for MOG_35−55_ from 2D2 mice. Anti-PD-L1 MAb was added in some co-cultures. In comparison with moDCs from control mice, moDCs from MOG_35−55_-tolerized mice induced lower T cell proliferation and lower GM-CSF and IL-17A production, but greater IFN-γ and PD-1 expression and annexin V staining by T cells (Supplementary Figures 1G–I). We also measured a larger quantity of regulatory cytokines, IL-27 and IL-10, in supernatants of these co-cultures compared with controls (Supplementary Figure 1J). In the presence of anti-PD-L1 MAb, moDCs did not reduce T cell proliferation or induce their apoptosis, demonstrating that PD-L1 expressed by moDCs limits myelin-specific CD4^+^ T cell responses *in vitro*.

To validate the immunosuppressive phenotype of MOG_35−55_/i.v.-induced moDCs *in vivo*, we transplanted moDCs from the CNS of tolerized mice with EAE into mice with ongoing EAE. Control mice received either moDCs from PBS/i.v. mice, PBS, or moDCs from MOG_35−55_/i.v. mice that were pretreated for 1 h with anti-PD-L1 MAb. The transfer of moDCs from MOG_35−55_-treated donors, but not from those that were PBS-treated, led to recovery from the disease, and anti-PD-L1 MAb pretreatment of moDCs from MOG_35−55_/i.v. donors precluded their suppressive effect on EAE in recipient mice (Figure 1M). These data show that moDCs of MOG_35−55_-treated mice with EAE are suppressive *via* PD-L1 *in vitro* and *in vivo*.

### Interferon-γ Secreted by CD4^+^ T Cells Is Necessary for Experimental Autoimmune Encephalomyelitis Suppression Upon Myelin Oligodendrocyte Glycoprotein_35−55_/Intravenous Treatment

**Figure 2:**
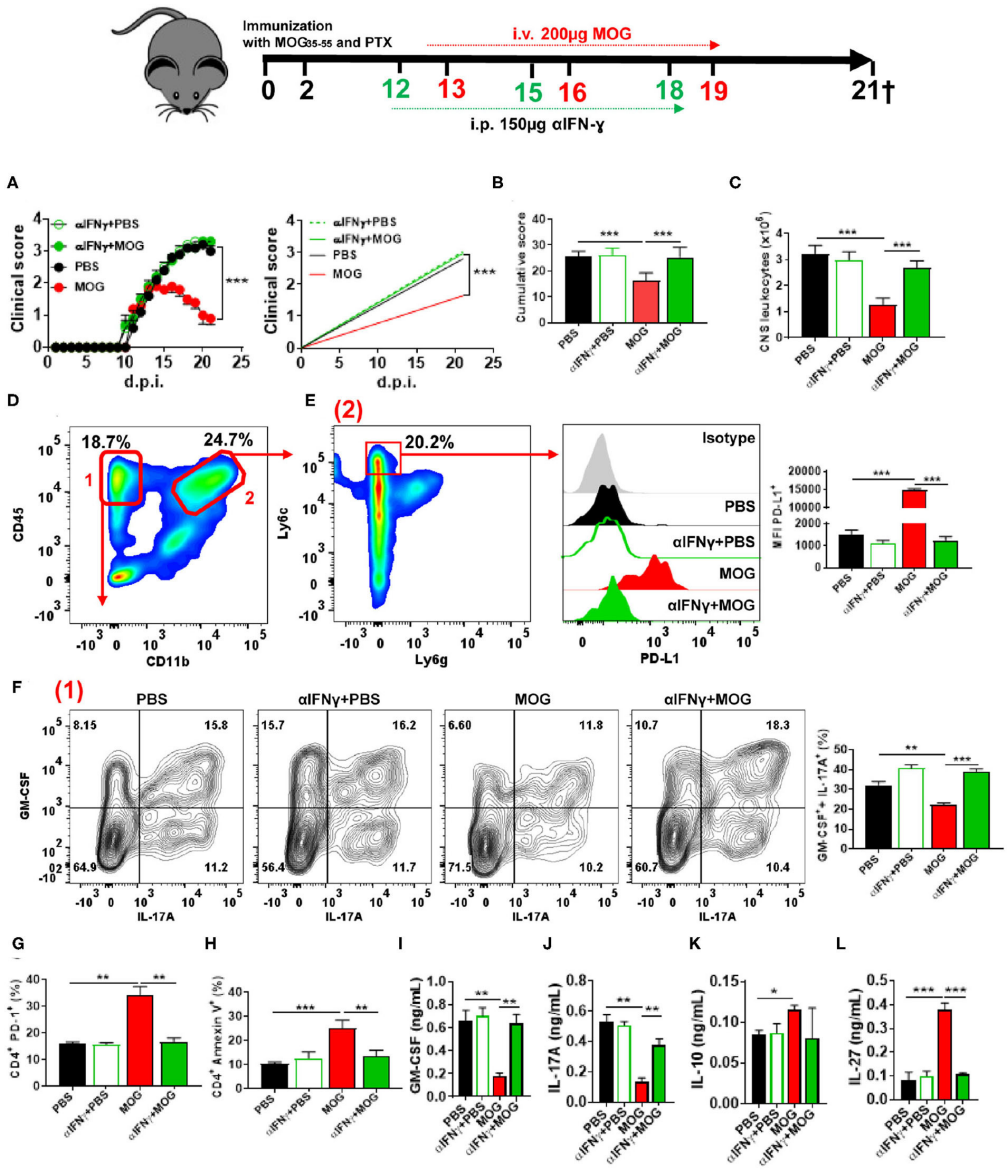
IFN-γ is critical for i.v. tolerance induction in EAE. **(A)** Mice with EAE (*n* = 5–7/group in each experiment) were i.p. injected with blocking anti-IFN-γ MAb (150 μg/mouse/injection; clone H22), or isotype control MAb, on 12, 15, and 18 d.p.i. MOG_35−55_ or PBS was i.v. given on 13, 16, and 19 d.p.i. **(B)** Cumulative disease score for mice described in **(A)**. **(C)** Mice described in **(A)** were sacrificed on day 21 p.i. and the numbers of CD45^+^ leukocytes in their CNS were determined by flow cytometry. **(D)** Flow cytometry plot showing lymphoid (1) and infiltrating myeloid (2) cells from the CNS of mice shown in **(A)**. **(E)** MFI of PD-L1^+^ moDCs from the CNS of mice described in **(A)**. Frequencies of GM-CSF^+^ IL-17A^+^ **(F)**, PD-1^+^ **(G)**, and annexin V^+^ **(H)** CD4^+^ T cells from the CNS of mice described in **(A)**. **(J–M)** Cytokine concentrations in supernatants from cultures of spleen cells from mice described in **(A)** stimulated for 72 h with MOG_35−55_. Cytokine concentrations were measured by ELISA. All data are representative of at least two experiments, and symbols depict mean ± SEM. Analysis between four groups was carried out by one-way ANOVA with Bonferroni post-test. EAE experiments in **(A)** were analyzed by two-way ANOVA with Bonferroni's multiple comparison. Values of *P < 0.05, **P < 0.001, and ***P < 0.0001 were considered significant.

**Figure 3:**
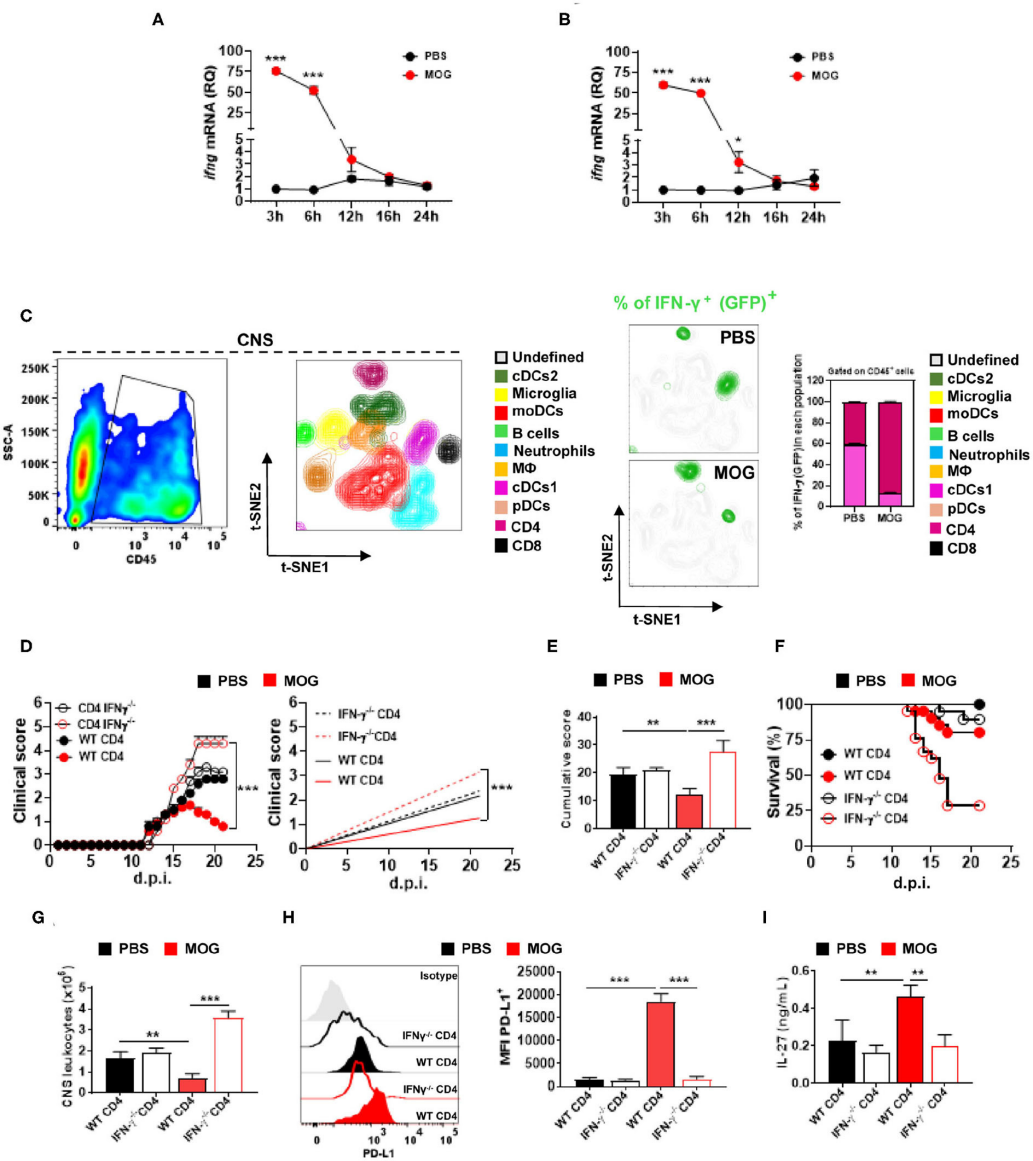
CD4^+^ T cell-derived IFN-γ is critical for i.v. tolerance induction in EAE. Time course of *Ifng* mRNA, analyzed by qPCR, in the spleen **(A)** and CNS **(B)** after treating mice with EAE i.v. with MOG_35−55_ peptide (200 μg) or PBS (*n* = 5/group in each experiment). **(C)** GFP**/**IFN-γ reporter mice were immunized for EAE induction and i.v. injected with MOG_35−55_ or PBS at disease onset (*n* = 5/group in each experiment). Mice were sacrificed 3 h post injection and CD45^+^ cells from the CNS were analyzed by flow cytometry. t-SNE graphs showing the expression and percentage of IFN-γ^+^ (GFP^+^) among these cells. Ten populations were identified: microglia (CD11b^+^ CD45^low^), monocytes (CD11b^+^ CD11c^+^ Ly6c^high^ MHCII^high^ Ly6g^−^), macrophages (CD11b^+^ Ly6c^low^ Ly6g^−^ MHCII^+^ F4/80^high^), cDC1 (CD11b^+^ CD11c^+^ Ly6c^med^ MHCII^high^ CD26^+^), cDC2 (CD11b^+^ CD11c^+^ Ly6c^med^ MHCII^+^ CD172α^+^), pDCs (Lin^−^ PDCA1^high^), B cells (CD19^+^ MHCII^+^), CD8^+^ T cells (CD11b^−^ CD3^+^ CD8α^+^), and CD4^+^ T cells (CD11b^−^ CD3^+^ CD4^+^). **(D)** RAG1^−/−^ mice were reconstituted with 5 × 10^6^ total CD4^+^ T cells from WT or IFN-γ^−/−^ mice (*n* = 10/group in each experiment); 72 h post reconstitution, recipient mice were immunized for EAE induction and MOG_35−55_ or PBS was given i.v. three times, starting at EAE onset. **(E)** Cumulative disease score for mice shown in **(D)**. **(F)** Survival (%) of EAE mice treated described in **(D)**. **(G)** Mice described in **(D)** were sacrificed 21 d.p.i. and the numbers of CD45^+^ leukocytes in their CNS were determined by flow cytometry. **(H)** MFI of PD-L1^+^ moDCs from the CNS of mice shown in **(D)**. **(I)** Splenocytes from mice described in **(D)** were stimulated with MOG_35−55_ for 72 h. Concentrations of IL-27 in culture supernatants were measured by ELISA. All data are representative of at least two experiments, and symbols depict mean ± SEM. Analyses between two groups were carried out by Student's *t*-test, and analyses between four groups by one-way ANOVA with Bonferroni post-test. EAE experiments in **(D)** were analyzed by two-way ANOVA with Bonferroni's multiple comparison. Values of *P < 0.05, **P < 0.001, and ***P < 0.0001 were considered significant.

Given that IFN-γ suppresses EAE (28, 34) and induces PD-L1 expression (35), we tested whether IFN-γ plays a role in tolerance induction. We injected anti-IFN-γ MAb into mice after EAE onset, 24 h before MOG_35−55_/i.v. injection (Figure 2A). Anti-IFN-γ-treated mice developed a severe disease that did not respond to MOG_35−55_/i.v. treatment (Figures 2B,C). These mice had markedly increased numbers of leukocytes in the CNS compared with control MOG_35−55_/i.v.-treated mice (Figure 2D). Anti-IFN-γ-treated mice also had lower expression of PD-L1^+^ in moDCs, reduced numbers of apoptotic CD4^+^ T cells, and greater frequencies of GM-CSF^+^ IL-17A^+^ CD4^+^ T cells (Figures 2E–I). Consistent with this observation, we found higher concentrations of GM-CSF and IL-17A in culture supernatants from MOG_35−55_-stimulated splenocytes of the above anti-IFN-γ-treated mice compared with controls (Figures 2J,K); however, while MOG_35−55_/i.v. treatment resulted in an increase of IL-10, and especially IL-27 production, treatment with anti-IFN-γ MAb precluded these increases (Figures 2L,M). We next determined the kinetics of IFN-γ expression upon MOG_35−55_ injection into mice with EAE. *Ifng* mRNA levels, both in the CNS and spleen, were highest at 6 h after the injection and declined to base levels by 12 h post injection (Figures 3A,B). These data show that IFN-γ plays a critical role in the induction of i.v. tolerance in EAE.

To identify the cellular sources of MOG_35−55_/i.v.-induced IFN-γ, we induced EAE in IFN-γ reporter mice (express GFP from the IFN-γ gene), injected them with MOG_35−55_/i.v. at disease onset, and 3 h after the injection analyzed their CNS. IFN-γ was primarily produced by CD4^+^ T cells, whereas in control mice injected with PBS, both cDCs and CD4^+^ T cells produced IFN-γ (Figure 3C). We then investigated whether CD4^+^ T cells are a relevant source of IFN-γ in tolerance induction. To this end, we reconstituted RAG1^−/−^ mice with WT or IFN-γ^−/−^ CD4^+^ T cells and immunized them for EAE induction; mice received PBS or MOG_35−55_/i.v. at disease onset. MOG_35−55_/i.v. significantly suppressed disease in mice with WT CD4^+^ T cells, whereas it exacerbated the disease in mice with IFN-γ^−/−^ T cells (70% of mice died) (Figures 3D,E). Consistent with clinical outcome, mice with IFN-γ^−/−^ T cells had reduced PD-L1^+^ expression in CNS moDCs compared with mice with WT T cells (Figure 3H). Moreover, splenocytes of mice with IFN-γ^−/−^ T cells upon *in vitro* activation produced significantly less IL-27 compared with control mice (Figure 3I). These data show that IFN-γ secretion by CD4^+^ T cells is required for EAE suppression by MOG_35−55_/i.v. treatment.

### The Lack of Interferon-γ Signaling in Conventional Dendritic Cells Type 1 Precludes Their Interleukin-27 Expression in Intravenous Tolerance Induction in Experimental Autoimmune Encephalomyelitis

**Figure 4:**
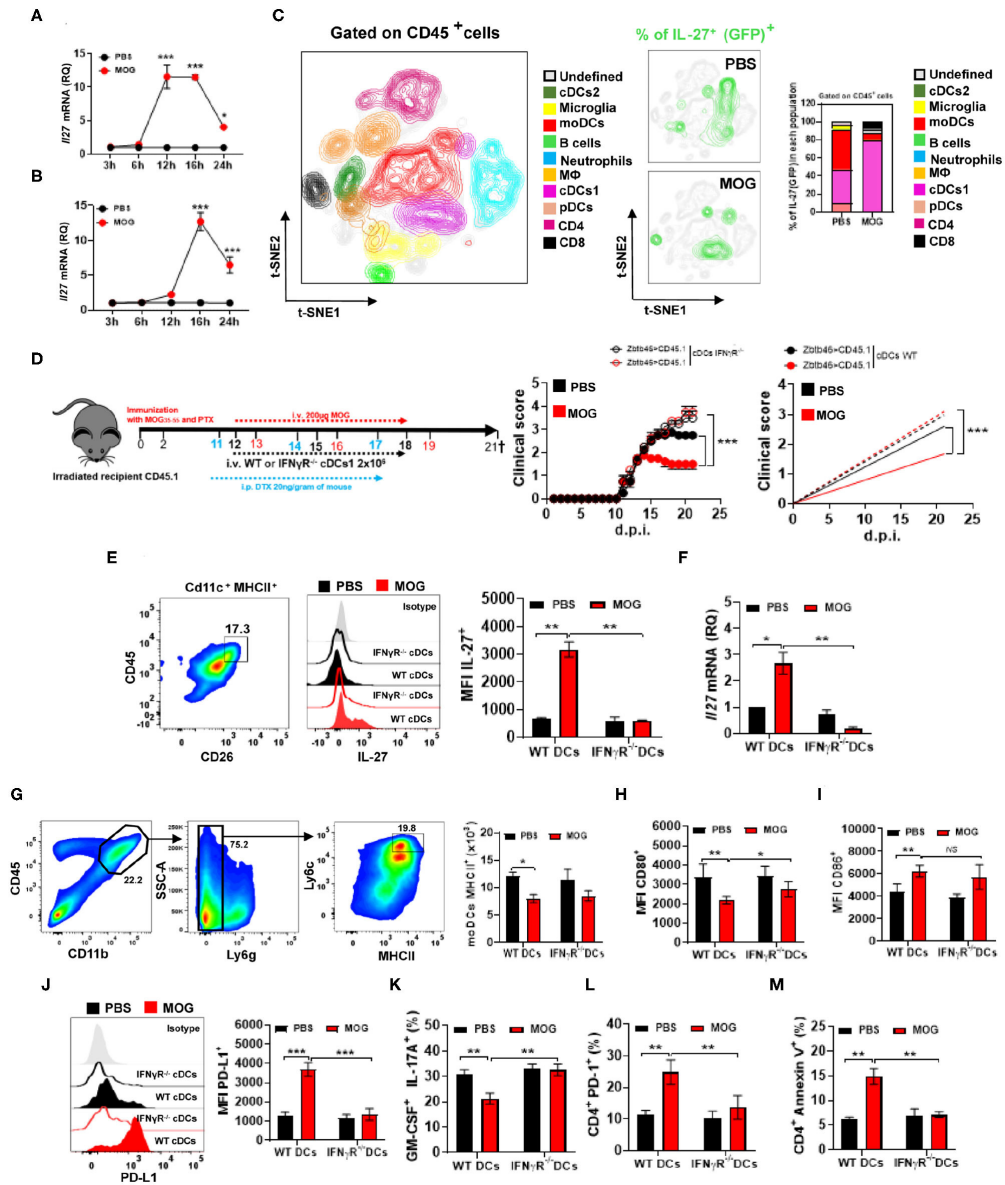
The lack of IFN-γ signaling in cDC1 abrogates their IL-27 production upon i.v. tolerance induction in EAE. Time course of *Il27* mRNA expression analyzed by qPCR, in the spleen **(A)** and CNS **(B)** 24 h after treating mice with EAE i.v. with MOG_35−55_ or PBS (*n* = 5/group in each experiment). **(C)** GFP**/**IL-27p28 reporter mice were immunized with MOG_35−55_ for EAE induction and i.v. injected with MOG_35−55_ or PBS at disease onset (*n* = 5/group in each experiment). Mice were sacrificed 16 h post injection and cells from the CNS analyzed by flow cytometry. Ten CD45^+^ cell populations were identified: microglia (CD11b^+^ CD45^low^), monocytes (CD11b^+^ CD11c^+^ Ly6c^high^ MHCII^high^ Ly6g^−^), macrophages (CD11b^+^ Ly6c^low^ Ly6g^−^ MHCII^+^ F4/80^high^), cDC1 (CD11b^+^ CD11c^+^ Ly6c^med^ MHCII^high^ CD26^+^), cDC2 (CD11b^+^ CD11c^+^ Ly6c^med^ MHCII^+^ CD172α^+^), pDCs (Lin^−^ PDCA1^high^), B cells (CD19^+^ MHCII^+^), CD8^+^ T cells (CD11b^−^ CD3^+^ CD8α^+^), and CD4^+^ T cells (CD11b^−^ CD3^+^ CD4^+^). t-SNE graphs show the expression and percentage of IL-27p28^+^ (GFP^+^) cells. **(D)** CD45.2^+^ mice were irradiated and transplanted with Zbtb46-iDTR or CD45.1^+^ BM and 6–8 weeks later immunized with MOG_35−55_ (*n* = 7–10/group in each experiment)_._ cDC depletion (Zbtb46^+^ MHCII^+^ CD11c^+^) was accomplished by i.p. injecting DTX (20 ng/g of mouse weight) every third day after EAE onset. *In vitro* Flt-3-differentiated BMDCs, IFN-γRα^−/−^, or WT was i.v. transferred 1 day post DTX injection, in total twice. MOG_35−55_ or PBS was i.v. injected at 13, 16, and 19 d.p.i. **(E)** At 21 d.p.i, cDC1 (CD11b^+^ CD11c^+^ CD45^+^ Ly6c^med^ MHCII^high^ CD26^+^) were isolated from the CNS of EAE mice described in **(D)** and their IL-27p28 production was analyzed by flow cytometry and **(F)** by qPCR. The numbers of MHCII^+^ **(G)**, CD80^+^ **(H)**, CD86^+^ **(I)**, and MFI of PD-L1^+^ **(J)** in moDCs from the CNS of mice described in **(D)**. Frequencies of GM-CSF^+^ IL-17A^+^ **(K)**, PD-1^+^ **(L)**, and annexin V^+^ **(M)** CD4^+^ T cells from the CNS of mice described in **(D)**. All data are representative of at least two experiments, and symbols depict mean ± SEM. Analysis between two groups was carried out by Student's *t*-test, whereas analysis between four groups was carried out by one-way ANOVA with Bonferroni post-test. EAE experiments in **(D)** were analyzed by two-way ANOVA with Bonferroni's multiple comparison. Values of *P < 0.05, **P < 0.001, and ***P < 0.0001 were considered significant.

Blocking IFN-γ resulted in reduced IL-27 production, prompting us to search for the cellular source of IL-27 upon MOG_35−55_/i.v. treatment. We induced EAE in IL-27 reporter mice (express GFP from the IL-27p28 gene) and injected them with MOG_35−55_ at disease onset. IL-27 production in the spleen and CNS peaked at 16 h after the injection (Figures 4A,B), and among cells from the CNS, the primary IL-27-producing cells were cDC1 (~80% of GFP^+^ cells), while in PBS-treated mice, moDCs also produced IL-27 (Figure 4C).

We next investigated whether the lack of IFN-γ signaling in cDC1 affects IL-27 production and compromises tolerance induction. We generated a Zbtb46-DTR (CD45.2^+^)→ CD45.1^+^ bone marrow (BM) chimera mice in which cDC1 can be depleted with diphtheria toxin (DTX) (36) (Supplementary Figure 2A). WT (CD45.2^+^)→ CD45.1^+^ BM chimeras served as control. We immunized chimera mice for EAE induction and depleted cDC1 by administering DTX, starting at disease onset and then every other day. We then transplanted *in vitro* Flt3-differentiated WT or IFN-γR^−/−^ cDC1 into these DTX-treated chimera mice with EAE (Figure 4D). Mice were treated with DTX and transplanted with cDC1 twice. Moreover, 24 h post cDC1 transplantation, mice were injected with PBS or MOG_35−55_, three times, 3 days apart. MOG_35−55_/i.v. treatment had no effect in mice that received IFN-γR^−/−^ cDC1, whereas in mice with WT cDC1, it significantly suppressed disease (Figure 4D). We also found a reduced IL-27 production (both mRNA and protein) by CNS IFN-γR^−/−^ cDC1 (Figures 4E,F), suggesting that IFN-γ induces IL-27 expression in cDC1. We next investigated whether reduced IL-27 production by IFN-γR^−/−^ cDC1 affected PD-L1 expression by moDCs and encephalitogenic CD4^+^ T cells. CNS moDCs from IFN-γR^−/−^ cDC1-transplanted mice did not differ in MHCII, CD80, and CD86 expression from moDCs of control mice, but they had significantly fewer PD-L1^+^ moDCs (Figures 4G–J). Failure of tolerance induction in IFN-γR^−/−^ cDC1-transplanted mice was associated with increased frequencies of IL-17^+^ and GM-CSF^+^ CD4^+^ T cells in the CNS (Figure 4K) and reduced numbers of annexin V^+^ and PD-1^+^ CD4^+^ T cells (Figures 4L,M) compared with WT cDC1-transplanted mice.

We then investigated whether, upon MOG_35−55/_i.v. injection, CNS and splenic cDC1 uptake MOG_35−55_, enabling them to directly interact with MOG_35−55_-specific T cells. Two hours post MOG_35−55_ or PBS i.v. injection into mice with ongoing EAE, cDC1 were FACS-sorted from the CNS (Supplementary Figures 2B,C) and co-cultured with 2D2 CD4^+^ T cells. cDC1 from MOG_35−55_-injected mice induced greater T cell proliferation and IFN-γ production, compared with cDC1 from PBS-injected mice (Supplementary Figures 2D–G). Overall, these data show that cDC1 acquire i.v. injected MOG_35−55_, which enables them to activate T cells and induce IFN-γ secretion. IFN-γ signaling in cDC1 is required to induce their IL-27 production, PD-L1 expression by moDCs, and i.v. tolerance in EAE.

### Interleukin-27 Induces Programmed Death Ligand 1 Expression in Monocyte-Derived Dendritic Cells

**Figure 5:**
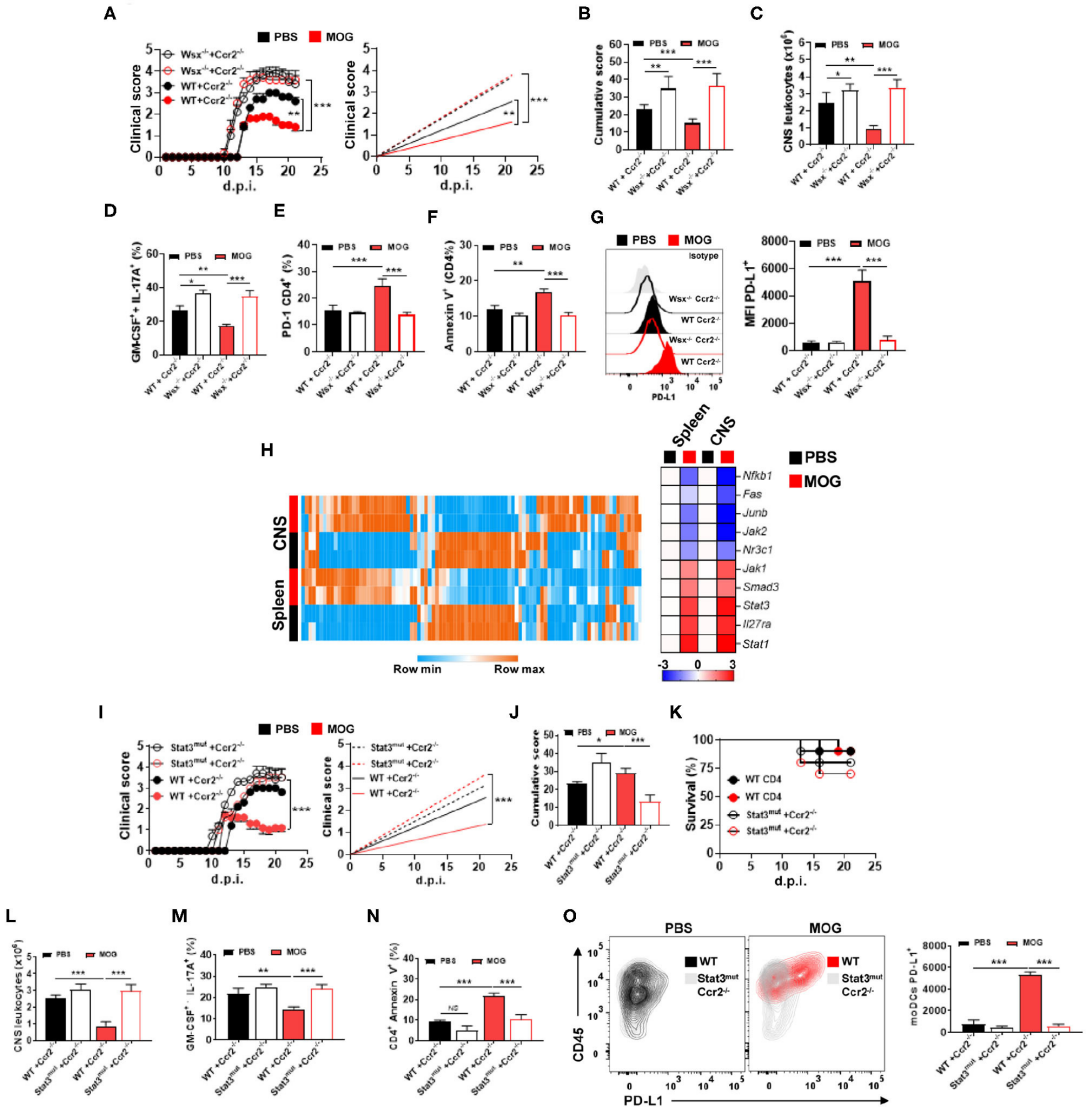
IL-27 induces PD-L1 expression in moDCs *via* STAT3. **(A)** CD45.1^+^ mice were irradiated and transplanted with 1:1 Wsx^−/−^ and *Ccr2*^−/−^ BM or WT and *Ccr2*^−/−^ BM and 6–8 weeks later immunized with MOG_35−55_ (*n* = 7–10/group in each experiment). Starting from disease onset, mice were i.v. injected with 200 μg of MOG_35−55_ every 3 days. **(B)** Cumulative disease score for mice shown in **(A)**. **(C)** Mice described in **(A)** were sacrificed at day 21 p.i. and the numbers of CD45^+^ leukocytes in their CNS were determined by flow cytometry. Frequencies of GM-CSF^+^ IL-17A^+^ **(D)**, PD-1^+^ **(E)**, and annexin V^+^ **(F)** CD4^+^ T cells from the CNS of mice shown in **(A)**. **(G)** MFI of PD-L1^+^ moDCs from the CNS of mice shown in **(A)**. **(H)** Splenic and CNS Ly6c^high^ MHCII^+^ monocytes were FACS-sorted from WT mice with EAE at 21 d.p.i. and Jak/Stat signaling gene array analysis was performed by qPCR. Heat map showing the expression levels of the top 10 genes. Gene expression levels are row centered, log2 transformed, and saturated at −3 and +3 for visualization. **(I)** CD45.1^+^ mice were irradiated and transplanted with 1:1 *Stat3*^*mut*^ and *Ccr2*^−/−^ BM, or WT and *Ccr2*^−/−^ BM, and 6–8 weeks later immunized with MOG_35−55_ (*n* = 7–10/group in each experiment). Starting from disease onset, mice were i.v. injected with 200 μg of MOG_35−55_ every 3 days. **(J)** Cumulative disease score for mice shown in **(I)**. **(K)** Survival (%) of mice treated as described in **(I)** (*n* = 10/group in each experiment). **(L)** Mice were sacrificed at day 21 p.i. and the numbers of CD45^+^ leukocytes from the CNS were determined by flow cytometry. Frequencies of GM-CSF^+^ IL-17A^+^ **(M)** and annexin V^+^ **(N)** CD4^+^ T cells in the CNS of mice shown in **(I)**. **(O)** Numbers of PD-L1^+^ moDCs from the CNS of mice shown in **(I)**. All data are representative of at least two experiments, and symbols depict mean ± SEM. Analysis between four groups was carried out by one-way ANOVA with Bonferroni post-test. EAE experiments **(A,I)** were analyzed by two-way ANOVA with Bonferroni's multiple comparison. Values of *P < 0.05, **P < 0.001, and ***P < 0.0001 were considered significant.

Given that IL-27 signaling is critical for i.v. tolerance induction (3) and that *Il27ra*^−/−^ (Wsx-1^−/−^) mice with EAE had reduced numbers of PD-L1^+^ moDCs in the CNS upon MOG_35−55_/i.v. treatment, we investigated whether the lack of IL-27 signaling in moDCs affects their PD-L1 expression and tolerance induction. We generated a mixed BM chimeras in which recipient mice (CD45.1) received half BM from *Ccr2*^−/−^ mice and another half from *Il27ra*^−/−^ (CD45.2) mice. In these chimera mice, virtually all monocytes outside of the BM are *Il27ra*^−/−^*Ccr2*^+/+^, as *Ccr2*^−/−^ monocytes fail to leave the BM (37). As a control, we generated mixed chimeras with BMs (1:1) from WT and *Ccr2*^−/−^ mice. Chimera mice were immunized for EAE induction and treated with MOG_35−55_/i.v. at disease onset. Mice with *Il27ra*^−/−^ BM developed severe disease, compared with mice with WT BM, and MOG_35−55_/i.v. treatment failed to suppress disease (Figures 5A,B). Worsening of disease and treatment failure were associated with a higher number of CNS-infiltrating leukocytes (Figure 5C) and greater frequencies of Th1 and Th17 cells, whereas the frequency of apoptotic CD4^+^ T cells was reduced (Figures 5D–F). Moreover, the lack of IL-27 signaling in CNS moDCs precluded upregulation of PD-L1 on them upon MOG_35−55_/i.v. treatment (Figure 5G). Taken together, these data show that MOG_35−55_/i.v.-induced IL-27 in turn induces PD-L1 expression in CNS moDCs.

CNS and spleen moDCs upregulated PD-L1 under MOG_35−55_/i.v. treatment, expressing high levels of genes related to Jak–Stat signaling (Figure 5H), especially signal transducer and activator of transcription-1 (STAT1) and STAT3. We therefore investigated whether they were involved in IL-27-mediated PD-L1 induction in moDCs. WT, *Stat1*^−/−^, and *Stat3*^mut^ [mutant STAT3 gene with impaired activity (38)] BM-derived DCs (BMDCs) were treated with IL-27 for 24 h, and PD-L1 expression was evaluated. While IL-27 treatment induced PD-L1 expression in WT and *Stat1*^−/−^ moDCs, *Stat3*^mut^ moDCs failed to upregulate PD-L1. Next, when cultured with naive 2D2 T cells, IL-27-treated *Stat3*^mut^ BMDCs were less suppressive to T cell proliferation and GM-CSF production, compared with IL-27-treated WT BMDCs.

To test whether STAT3 is necessary for PD-L1 induction in moDCs *in vivo*, we generated mixed BM chimera mice in which recipient mice (CD45.1) received BM cells from *Ccr2*^−/−^ mice and from *Stat3*^mut^ mice. In these mice, virtually all monocytes and monocyte-derived cells (e.g., moDCs) outside of BM are *Stat3*^mut^, whereas other immune cells are approximately 1:1 mixture of *Stat3*^mut^ and *Stat3*^WT^ cells. Control chimera mice received BM cells from *Ccr2*^−/−^ and WT mice; in these mice, monocytes and moDCs outside of the BM are *Stat3*^WT^. Chimera mice were immunized for EAE induction and, after onset of clinical disease, treated with MOG_35−55_/i.v. Mice with *Stat3*^mut^ BM developed severe EAE and did not respond to MOG_35−55_/i.v. treatment (Figures 5I,J). Treatment failure was associated with an increased number of CNS-infiltrating leukocytes (Figure 5L), higher frequencies of CNS-infiltrating Th1 and Th17 cells (Figure 5M), reduced frequency of apoptotic CD4^+^ T cells (Figure 5N), and reduced PD-L1^+^ expression in moDCs (Figure 5O). Moreover, although *Ccr2*^−/−^ *Stat3*^mut^ chimera mice developed severe EAE, we did not find statistical differences in survival compared with *Ccr2*^−/−^ WT mice (Figure 5K). These findings show that IL-27 induces PD-L1 expression in moDCs *via* the STAT3 pathway.

## Discussion

Extinguishing harmful immune responses by restoring peripheral tolerance toward auto-Ags has been a long-standing goal in the search for therapies for autoimmune diseases (1, 39). Although depletion of autoreactive T cells and induction of Tregs and tolerogenic DCs are well-known mechanisms of peripheral tolerance (5, 40, 41), our study defines an interplay between molecular and cellular factors that leads to the development of tolerogenic DCs and depletion of autoreactive T cells. Auto-Ag administered i.v. is acquired by cDC1 and presented to auto-Ag-specific T cells, leading to their activation and IFN-γ secretion, which in turn induces IL-27 secretion from cDC1. IL-27 acts on moDCs to induce PD-L1 expression, which then promotes apoptosis of PD-1^+^ autoreactive T cells and disease amelioration.

It has been established that IFN-γ plays a protective role in EAE through multiple mechanisms (22, 25, 42, 43); it is therefore not surprising that it also mediates disease suppression in i.v. tolerance induction. We have shown that i.v. tolerized mice with EAE have higher frequencies of IFN-γ^+^ CD4^+^ T cells compared with controls (3). We show here that i.v. injection of auto-Ag in mice with EAE induces a robust and rapid production of IFN-γ by CD4^+^ T cells and that blockade of IFN-γ inhibits IL-27 production and PD-L1 expression in CNS moDCs and abrogates tolerance induction. This is in agreement with the findings that IFN-γ prevents accumulation of activated CD4^+^ T cells in response to Ag stimulation by both inhibiting proliferation and inducing apoptosis of CD4^+^ T cells (25). Rapid *in vivo*/*in situ* IFN-γ secretion by peptide-specific effector memory CD4^+^ T cells upon i.v. injection of the peptide has been demonstrated (44), a finding fully applicable to our system. Thus, our results demonstrate that IFN-γ derived from CD4^+^ T cells is critical for i.v. tolerance induction in ongoing EAE.

Several studies have reported that distinct DC subpopulations can uptake Ags and induce immune tolerance by the induction of IL-27 production and Tregs (3, 10, 45). We have shown that CD11b^+^ CD103^−^ DCs are the major source of IL-27 in i.v. tolerance induction in EAE (3). The engagement of IFN-γR on DCs induces their expression of IL-27 and several other regulatory molecules, and IFN-γ-modified DCs modulate EAE severity in an IL-27-dependent manner (42). DCs treated with IFN-γ *in vitro* and injected into mice with EAE suppress disease (46). Consistent with this, we show here that mice lacking IFN-γ signaling in cDC1 fail to recover from EAE upon auto-Ag i.v. treatment. We also show that cDC1 uptake i.v. injected myelin Ag activate CD4^+^ T cells and their IFN-γ expression and induce tolerance.

The role of monocytes in EAE is viewed as solely pro-inflammatory (47, 48). However, there is evidence that CNS moDCs can acquire regulatory phenotype and facilitate tissue repair (49). Given that IL-27 induces PD-L1 in moDCs (8, 50) and that IL-27 signaling is beneficial in EAE (10, 51), we investigated whether the absence of IL-27 signaling in moDCs affects tolerance induction. We show that the absence of IL-27R in CNS moDCs abrogates the expression of PD-L1 and EAE recovery upon auto-Ag i.v. treatment. In contrast, neutrophils do not upregulate PD-L1 upon injection of auto-Ag; instead, they upregulate PD-L2, which is dispensable for tolerance induction.

It is well-known that IL-27 induces IL-10 expression by T cells and other types of immune cells (10). We and others have shown that i.v. tolerance induction in EAE induces IL-10 production (2, 5), which was also the case in this study. Further, the essential role of IL-10 in i.v. tolerance induction in EAE has been clearly demonstrated (2, 3, 41). We therefore did not test its role again here; however, in future studies, it would be interesting to define a pathway by which IL-10 mediates i.v. tolerance, such as its relevant cellular sources and targets, and to determine which effects of IL-27, if any, are not reliant on IL-10 induction.

Mice lacking PD-L1 develop exacerbated EAE, with PD-L1 on CD11c^+^ DCs playing an important role in limiting self-reactive CD4^+^ T cells (52). However, the lack of PD-L2, also a PD-1 ligand, did not worsen EAE (53), demonstrating that PD-L1 has a dominant role in regulating EAE severity. In agreement with these findings, our data reveal that PD-L1 is required for tolerance induction, whereas PD-L2 is dispensable. This is seemingly at odds with studies showing that blockade of PD-L1 with MAb at chronic stage EAE in C57BL/6 mice does not worsen the disease, whereas blockade of PD-L2 does (16, 53). A possible reason for this inconsistency is that we induced i.v. tolerance while clinical disease was still developing, whereas the abovementioned studies started PD-L1 and PD-L2 blockade later, in the chronic phase of disease. Taken together, these findings suggest that the relative importance of cell types and factors they express in regulating disease does change over the disease course.

Studies have shown that IL-27 induces PD-L1 expression (8) and that STAT3, which together with STAT1 mediates IL-27R signaling (3, 10), is required for PD-L1 expression (12, 54). However, the intracellular pathways downstream of IL-27 in i.v. tolerance induction in EAE are still unclear. Consistent with our previous finding that STAT1 is not necessary for IL-27-induced DC modulation (3), we show here that IL-27 from cDC1 induces PD-L1 expression in moDCs *via* STAT3. Indeed, BM chimera mice with impaired STAT3 signaling in moDCs failed to upregulate PD-L1 and to recover from EAE upon MOG_35−55_/i.v. treatment.

Our findings define the regulatory pathway that suppresses auto-Ag-specific immune response. The prerequisite for activation of this pathway is the existence of a large pool of auto-Ag-specific effector T cells that secrete IFN-γ upon activation with auto-Ag presented by APCs. Injection of a large quantity of auto-Ag induces a burst of IFN-γ secretion from auto-Ag-specific effector T cells, eliciting IL-27 and PD-L1 expression by APCs, which then in turn suppress immune response by causing anergy/apoptosis of the T cells. This is a regulatory feedback mechanism for dampening strong and possibly damaging immune responses. It is likely that this mechanism regulates myelin-specific autoimmune responses in EAE throughout its course, not only after i.v. tolerance induction. The existence of this pathway provides a unifying explanation for more severe disease in IFN-γ-, IL-27-, and PD-L1-deficient animals. It is likely that additional molecules participate in this pathway, such as IL-10, which is induced in and essential to i.v. tolerance induction in EAE (2, 3, 5, 55); upregulation of TGF-β has also been noted (55), but its significance not explored. Even though we have defined it in the context of i.v. tolerance induction in EAE, this regulatory pathway is certainly relevant in other contexts, being either beneficial or detrimental in them. In addition to CD4^+^ T cells, the source of IFN-γ could be, for example, pathogen-specific CD8^+^ T cells or NK cells as well.

## Data Availability Statement

The raw data supporting the conclusions of this article will be made available by the authors, without undue reservation.

## Ethics Statement

The animal study was reviewed and approved by Thomas Jefferson University.

## Author Contributions

GC and BC designed the concept and experiments and wrote the manuscript. GC performed most of the experiments. JR performed some of the flow cytometry experiments and revised the manuscript. RT, HD, AV, LI, and AB performed some *in vivo* experiments and helped with flow cytometry analysis. DH, WZ, and DX revised the manuscript. JP helped with the *in vivo* experiments and revised the manuscript. G-XZ revised the manuscript. JA conducted some *in vivo* experiments and revised the manuscript. AR and BC supervised the studies. All authors contributed to the article and approved the submitted version.

## Conflict of Interest

The authors declare that the research was conducted in the absence of any commercial or financial relationships that could be construed as a potential conflict of interest.
