# Supplementary figures and images for: Interferon-γ/Interleukin-27 Axis Induces Programmed Death Ligand 1 Expression in Monocyte-Derived Dendritic Cells and Restores Immune Tolerance in Central Nervous System Autoimmunity
Source: Front Immunol. 2020 Oct 26;11:576752. doi: 10.3389/fimmu.2020.576752 (PMC7649367; doi:10.3389/fimmu.2020.576752)

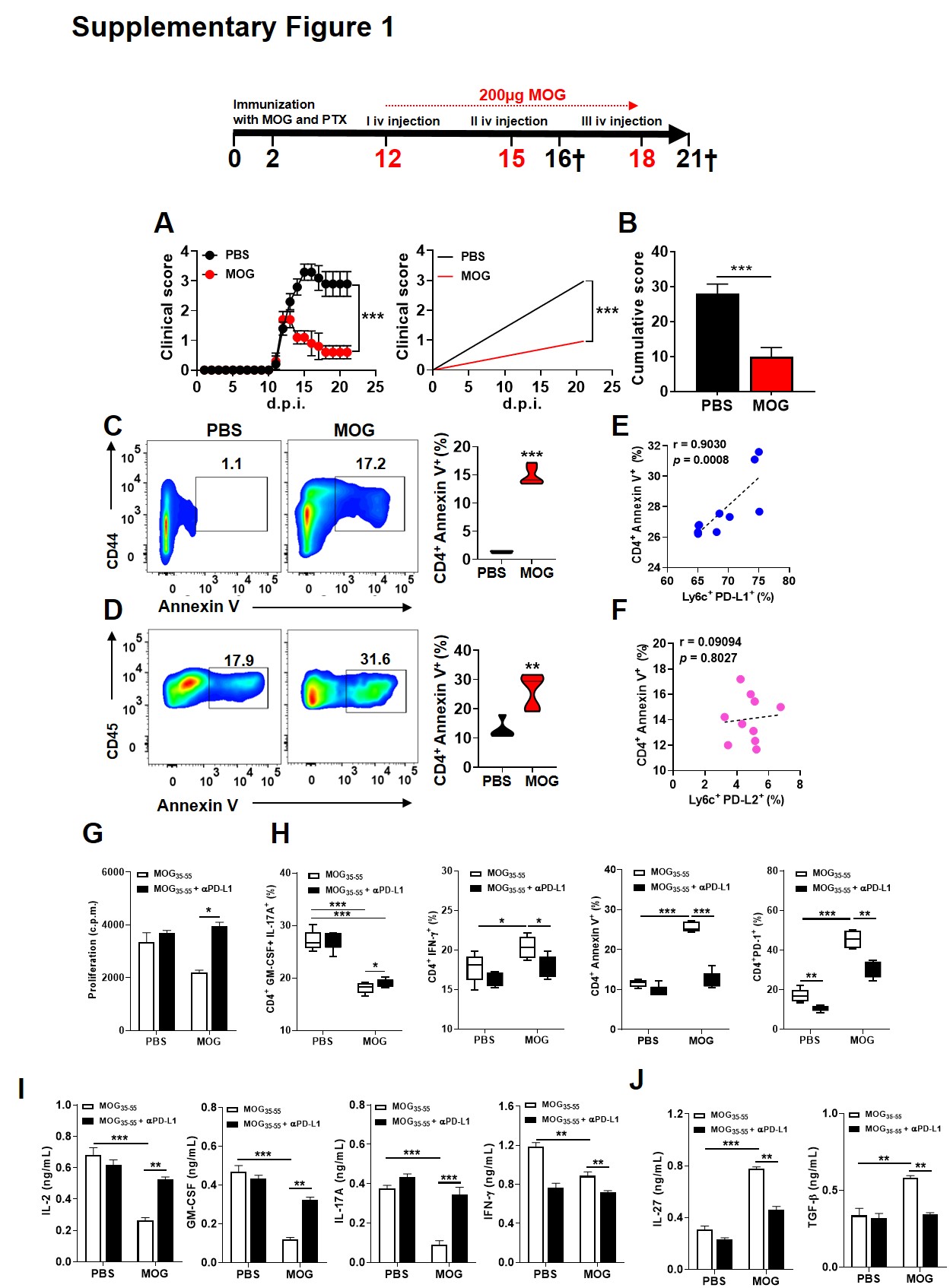

Supplement: Supplementary file 1 [file Image_1.jpeg]

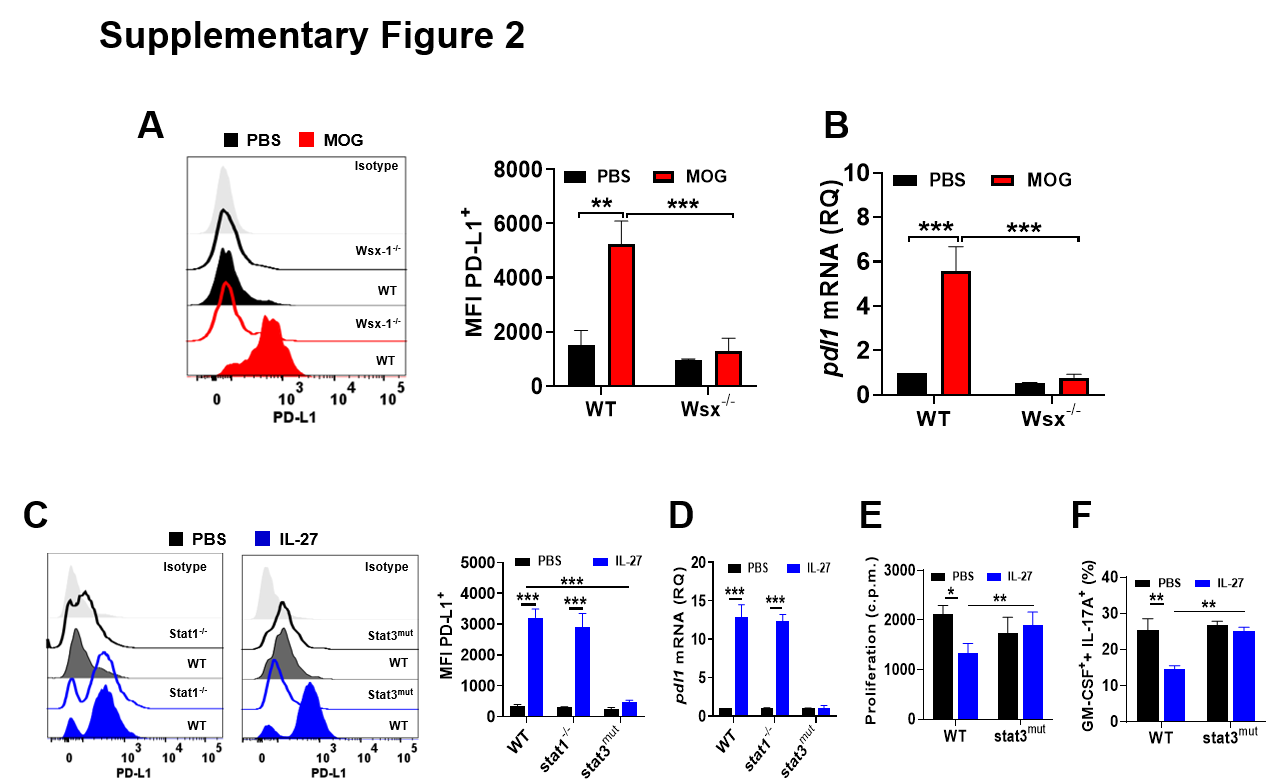

Supplement: Supplementary file 2 [file Image_2.tif]
